# Muscle and liver glycogen utilization during prolonged lift and carry exercise: male and female responses

**DOI:** 10.14814/phy2.13113

**Published:** 2017-02-27

**Authors:** Thomas B. Price, Kimberly Sanders

**Affiliations:** ^1^ Department of Diagnostic RadiologyYale University School of MedicineNew HavenConnecticut; ^2^ School of Arts and SciencesUniversity of BridgeportBridgeportConnecticut; ^3^ School of Naturopathic MedicineUniversity of BridgeportBridgeportConnecticut

**Keywords:** ^13^C‐NMR, lift/carry exercise, liver glycogen, menstrual cycle, muscle glycogen

## Abstract

This study examined the use of carbohydrates by men and women during lift/carry exercise. Effects of menstrual cycle variation were examined in women. Twenty‐five subjects (15 M, 10 F) were studied; age 25 ± 2y M, 26 ± 3y F, weight 85 ± 3 kg* M, 63 ± 3 kg F, and height 181 ± 2 cm* M, 161 ± 2 cm F (* *P* < 0.0001). During exercise subjects squatted to floor level and lifted a 30 kg box, carried it 3 m, and placed it on a shelf 132 cm high 3X/min over a 3‐hour period (540 lifts) or until they could not continue. Males were studied in a single session, females were studied on separate occasions (during the luteal (L) and follicular (F) menstrual phases). The protocol was identical for both sexes and on both occasions in the female group. Glycogen utilization was tracked with natural abundance C‐13 NMR of quadriceps femoris and biceps brachialis muscles, and in the liver at rest and throughout the exercise period. Males completed more of the 180 min protocol than females [166 ± 9 min M, 112 ± 16 min* F (L), 88 ± 16 min** F (F) (*P = 0.0036, **P < 0.0001)]. Quadriceps glycogen depletion was similar between sexes and within females in L/F phases [4.7 ± 0.8 mmol/L‐h M, 4.5 ± 2.4 mmol/L‐h F (L), 10.3 ± 3.5 mmol/L‐h F (F)]. Biceps glycogen depletion was greater in females [2.7 ± 0.9 mmol/L‐h M, 10.3 ± 1.3 mmol/L‐h* F (L), 16.8 ± 4.8 mmol/L‐h** F (F) (* *P* = 0.0004, ** *P* = 0.0122)]. Resting glycogen levels were higher in females during the follicular phase (*P* = 0.0077). Liver glycogen depletion increased during exercise, but was not significant. We conclude that with non‐normalized lift/carry exercise: (1) Based on their smaller size, women are less capable of completing and work their upper body harder than men. (2) Women and men work their lower body at similar levels. (3) Women store more quadriceps carbohydrate during the follicular phase. (4) The liver is not significantly challenged by this protocol.

## Introduction

Today many occupations for both men and women involve day‐to‐day lifting and carrying heavy objects. Jobs such as moving supplies, perishables, or merchandise can include daily extended periods of lift/carry work. Studies of exercise on consecutive days have observed that muscles, which rely on stored carbohydrates to provide fuel for day‐to‐day work may gradually deplete their energy reserves by undercompensating over several consecutive days of exercise and recovery (Costill et al. 1971; Kirwan et al. 1988; Pascoe et al. 1990). However, we recently observed a different pattern of daily muscle carbohydrate recovery over several consecutive days of prolonged lift/carry exercise in which exercised muscles overcompensated during each day‐to‐day recovery period (Price and Brady 2015). While this may be due simply to the difference in the exercise protocols employed in our study versus these previous studies (Costill et al. 1971; Kirwan et al. 1988; Pascoe et al. 1990; Price and Brady 2015), prolonged exercise on consecutive days has not been well‐studied and deserves further investigation. When a muscle with significantly reduced energy reserves is called upon to perform prolonged work, the body may redistribute the workload to different muscles (Selen et al. 2007). Hence, there is a need to understand how energy reserves are depleted during the period of exercise.

Adaptation of different movement patterns has been observed in women performing fatiguing repetitive exercise (Hunter et al. 2004a, 2004b; Ge et al. 2005; Selen et al. 2007; Qin et al. 2014), and can result in “less‐than‐optimal” biomechanical form which may lead to occupational injuries (Ge et al. 2005). Owing to gender differences in size, the upper body isometric mean lifting strength (MLS) of women is about 60% of men (Sharp 1994), suggesting that women who work in occupations requiring prolonged repetitive lifting may be at a greater risk of musculoskeletal injury, when working alongside men, under daily time constraints when a job needs to be done (Latash and Anson 1996; Ge et al. 2005; Madeleine et al. 2008). This study examines sex‐related differences in response to prolonged lift/carry exercise.

Previous studies have shown that specific patterns of carbohydrate depletion occur during prolonged exercise, and that these patterns are dependent upon workload (Costill DL et al. 1973; Gollnick et al. 1974; Vollestad and Blom 1985; Price et al. 1991, 1994a; Tesch et al. 1998). Early studies of running and cycling exercise demonstrated that the rate of glycogen depletion increased as workload was increased, also showing that initial depletion occurred almost entirely in slow twitch (ST) muscle fibers (Costill DL et al. 1973; Gollnick et al. 1974). A 1985 study observed depletion of almost all available glycogen in ST fibers and 20% of fast twitch type a (FTa) fibers following 1 h of cycling at 43% of maximum oxygen uptake volume (VO_2max_) (Gollnick et al. 1974). At 61% of VO_2max_ all of ST glycogen was depleted along with 65% of FTa glycogen after an hour, and the depletion rate was doubled (Vollestad and Blom 1985). A subsequent study utilizing resistance exercise produced results similar to studies that employed running and cycling, also demonstrating that as workloads peaked FT type b (FTb) fibers were utilized as well(Tesch et al. 1998). While the advantage of these studies, which obtained data from biopsy samples, is the ability to track glycogen depletion in individual muscle fiber types, the disadvantage is the limited number of times a biopsy can be performed during the exercise period and the inability to get multiple samples from the same tissue (Costill DL et al. 1973; Gollnick et al. 1974; Vollestad and Blom 1985; Tesch et al. 1998). So tracking the time course of glycogen depletion in exercising muscles during exercise is limited, if possible at all. Another method of obtaining muscle glycogen concentrations in vivo, natural abundance ^13^C‐NMR spectroscopy (^13^C‐MRS), is noninvasive and can obtain many measurements throughout a period of exercise (Price et al. 1991, 1994a). While this method can provide glycogen depletion patterns in specific exercising muscles during an exercise period, it cannot distinguish between fiber types. We have studied low‐intensity exercise in isolated human gastrocnemius muscles performing plantar flexion exercise at three different workloads establishing a pattern of initial glycogen depletion followed by a secondary period of no more net depletion despite up to 6 h of continuing exercise (Price et al. 1991).

Exercise performed on different days at different workloads [15, 20, and 25% of maximum voluntary contraction (MVC)] depleted about 20 mmol/kg‐ww of gastrocnemius glycogen within an approximately 11 mL volume of muscle (Price et al. 1991). However, the biphasic pattern of depletion followed by cessation of net depletion occurred more rapidly as the workload increased. Glycogen depletion rates were 4.8 mmol/kg‐hr at 15% MVC, 5.7 mmol/kg‐h at 20% MVC, and 7.5 mmol/kg‐h at 25% MVC (Price et al. 1991). Subsequently we employed an infusion of 100% enriched 1‐^13^C‐labeled glucose, observing glycogen turnover during the secondary exercise period of no net glycogen depletion (Price et al. 1994a).

When comparing responses to prolonged exercise in men and women, the hormonal variations that occur in the different phases of the female menstrual cycle must be considered. During the follicular (F) phase both estrogen (E) and progesterone (P) are low, whereas during the luteal (L) phase both levels rise (Oosthuyse 2010). These levels of E and P, as well as the ratio of the two levels (E/P), can exert multiple influences on carbohydrate metabolism during exercise (Jurkowski et al. 1981; Lavoie et al. 1987; Nicklas et al. 1989; DeSouza et al. 1990; McCracken et al. 1994; Bemben et al. 1995; Campbell et al. 2001; Zderic et al. 2001; Horton et al. 2002; Suh et al. 2002; Dean et al. 2003; Forsyth and Reilly 2005; Devries et al. 2006). While some studies comparing running or cycling exercise performed during the F versus L phases have shown a menstrual effect (Jurkowski et al. 1981; Lavoie et al. 1987; McCracken et al. 1994; Zderic et al. 2001; Forsyth and Reilly 2005), others have shown no significant effect (Nicklas et al. 1989; Bemben et al. 1995; Campbell et al. 2001; Horton et al. 2002, 2006; Suh et al. 2002; Dean et al. 2003). Several studies employed cycling at moderate to heavy workloads during the F phase producing blood lactate levels that were greater than in the L phase (Jurkowski et al. 1981; Lavoie et al. 1987; McCracken et al. 1994; Zderic et al. 2001), whereas in other studies running and cycling at moderate workloads failed to produce significant menstrual effects (Nicklas et al. 1989; DeSouza et al. 1990; Bemben et al. 1995; Campbell et al. 2001; Horton et al. 2002; Dean et al. 2003). This suggests that workload and type of exercise may play a role. Glucose turnover was also found to be greater with moderate exercise in the F phase (Lavoie et al. 1987; Zderic et al. 2001).

When cycling during the F phase, untrained women (VO_2max_ = 39 ml/kg‐min) working 90 min at 65% of VO_2max_ used 24% more muscle glycogen than when performing an identical protocol during the L phase (Devries et al. 2006). The increased levels of E during the L phase have been shown to prolong the time to exhaustion in cycling exercise (Jurkowski et al. 1981); however, E/P ratio is an important factor (Jurkowski et al. 1981; Nicklas et al. 1989; Devries et al. 2006). As observed in both humans and animals, the E/P ratio may then be the most important controlling factor in carbohydrate catabolism as well as fat mobilization (Benoit et al. 1982; Kendrick et al. 1987; Ellis et al. 1994; Hansen et al. 1996; Campbell and Febbraio 2002; Devries et al. 2006; Horton et al. 2006). Taken together, previous studies support the idea that female menstrual influences on carbohydrate catabolism during exercise is complex and subject to a number of influences including: (1) E/P ratio, (2) workload, (3) type of exercise, and (4) individual subject variability on any given day. Resistance exercise has been employed to a limited extent in studies of the effect of menstrual cycle on hormonal response (Kraemer et al. 1993; Nakamura et al. 2011) and strength (Loureiro et al. 2011), but not energy metabolism. While the majority of human data relating to menstrual effect upon carbohydrate utilization comes from cycling or running exercise, to our knowledge, no data exists on prolonged resistance exercise.

In this study, we sought to examine systemic metabolic responses to prolonged lifting and carrying exercise in male and female subjects. Responses were compared between sexes, and within the female group during different phases of the menstrual cycle. Glycogen depletion patterns were studied in two of the primary muscles utilized for lift/carry exercise, and hepatic glycogen depletion was measured with ^13^C‐MRS of the liver. We hypothesized that (1) Exercising muscle glycogen depletion patterns would differ between males and females, (2) Exercising muscle glycogen depletion patterns would differ within the female population between luteal and follicular phases of the menstrual cycle, and (3) Liver glycogen depletion would differ as a function of sex.

## Methods

### Subjects

Twenty‐five subjects (15M, 10F) were studied. Males (28 ± 2 yrs, 85 ± 3 kg, 181 ± 2 cm) and females (26 ± 3 yrs, 63 ± 3 kg*, 167 ± 2 cm*, *P* ≤ 0.0001 vs males) were matched for age and training, but not weight and height (Table 1). All subjects were nonsmokers. Fitness levels of male and female subjects, rated according to the Army Physical Fitness Test (Form DA 705), were not significantly different (Table 1). Composite percentiles were: 84 ± 7 percentile, sit‐ups; 92 ± 5 percentile, push‐ups; 96 ± 4 percentile, 2‐mile run, as compared with active soldiers. Subjects with a family history of diabetes and/or hypertension were excluded from the study, as were females currently on birth control. Females were studied during the luteal (L) (3‐6da pre‐menses) and follicular (F) (3‐6da post‐menses) phases of their menstrual cycles. Informed consent was obtained from all subjects following an explanation of the nature, the potential risks, and possible consequences of the study according to a protocol approved by the Human Investigation Committee of the Yale University School of Medicine.

**Table 1:** Subject characteristics male (*n* = 15) and female (*n* = 10) participants

Subject characteristics					
	Age (yrs)	Height (cm)	Weight (kg)	BMI	Percentile Army PRT
Male	28 ± 2	181 ± 3	85 ± 3	26.9 ± 0.8	90^th ^± 6^th^
Female	26 ± 3	167 ± 2*	63 ± 3*	23.0 ± 0.6**	94^th ^± 6^th^

*P < 0.0001 F vs. M, **P = 0.0008 F vs. M.

PRT percentiles, which represent level of fitness according to the US Army physical readiness test, indicate that these subjects were at a similar level of fitness as US soldiers.

### Exercise protocol

Subjects arrived at the Magnetic Resonance Research Center at 9AM on the day of the study, having been allowed to eat a liquid meal immediately upon waking (Ensure: 6 g fat, 9 g protein, 32 g carbohydrate, 220 kcal). Times from consumption of the meal to start of exercise were similar between groups (2.5 ± 0.2 h M, 2.3 ± 0.3 h FL, 2.5 ± 0.3 h FF). No further meals were allowed until the exercise session was completed.

**Figure 1:**
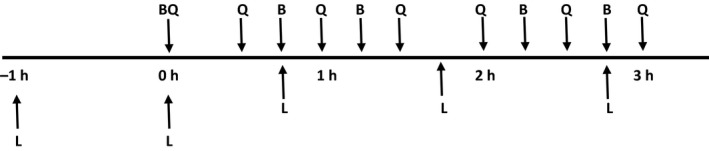
Timeline of 1‐13C MRS measurements before, during and after lift/carry exercise period. (L – Liver, B – Left Biceps Brachialis, Q – Left Quadriceps Femoris)

Baseline MRS measurements were made in the liver, the left quadriceps muscle group (vastus lateralis), and the left upper arm (biceps brachialis) during the initial 60 min prior the first 15 min bout of exercise (Fig. 1). During the exercise period MRS data were obtained from one of the three sites following each subsequent 15 min bout of exercise, rotating between sites (Fig. 1). This was necessitated because of the time required to position the subject and obtain a single MRS measurement (averaging 13–15 min).

Subjects were asked to squat and lift a weighted box (30 kg) from six inches above floor level, walk 3 meters carrying the box and place it on the upper end of an exercise ergometer at a height of 1.3 meters (Price and Brady 2015). Upon placement, the box travelled back to floor level and was lifted again. The exercise protocol, consisting of three hours of squat/lifts at three lifts per minute (one lift every 20 seconds) stopping every 15 min to perform MRS (12 15 min blocks of exercise, 540 total lifts), was not normalized for subject size or sex with both men and women being asked to perform identical tasks. The exercise protocol was continued until subjects either completed the task or could no longer continue to exercise. Mean heart rate was taken as the average of measurements collected every 5 min during each exercise period.

### Skeletal muscle MRS

Natural abundance ^13^C‐NMR (MRS) spectroscopy was performed at 2.1 T on a Bruker Biospecspectrometer with a 100‐cm‐diameter magnet bore (Maughan et al. 1983). During the measurements, subjects remained supine within the magnet with a surface coil radio‐frequency (RF) probe resting directly over the muscle to ensure that the majority of signal was received from the muscle of interest (vastus lateralis/intermedius or biceps brachialis). A microsphere containing a ^13^C‐labeled formate was fixed at the center of the RF coil for pulse widths. Subjects were positioned by an image‐guided localization routine employing a T_1_‐weighted gradient‐echo image (repetition time = 82 milliseconds, echo time = 21 milliseconds). Subjects were positioned so the isocenter of the magnetic field was approximately two centimeters into the muscle. By determining the 180^o^‐flip angles at the center of the observation coil from the microsphere standard, RF pulse widths were set so that the 90^o^‐pulse was sent to the center of the muscle.

This technique maximizes suppression of the lipid signal that arises from the subcutaneous fat layer and optimizes the signal from the muscle. The ^1^H‐decoupled ^13^C RF pulse sequence was designed so that 5472 summed ^13^C transients were obtained. The repetition time for ^13^C acquisition was 87 milliseconds, and ^1^H continuous wave decoupling was truncated to 25 milliseconds at the beginning of each ^13^C acquisition to prevent excessive RF power deposition in the muscle. During the data acquisition period, RF power was pulsed through the surface coil at a frequency of 22.5 MHz (^13^C resonance frequency). A 9‐centimeter diameter circular ^13^C surface coil RF probe was used for spectral acquisitions. Shimming, imaging, and ^1^H decoupling at 89.5 MHz was performed with a butterfly coil. Proton line widths are typically shimmed to 70 Hz. The total scan time for each spectrum was eight minutes. Skeletal muscle spectra were collected from the left vastus lateralis and left biceps brachialis. ^13^C spectra were then processed using a 30 Hz Lorentzian to Gaussian broadened filter, and baseline‐corrected to ±300 Hz on either side of the 1‐^13^C glycogen resonance of subject and sample spectra, and integrated areas were assessed ±100 Hz about the resonance and compared. Glycogen (GLY) concentrations were determined by comparison with an external standard solution (150 mmol/L glycogen + 50 mmol/L KCl) that loaded the RF coil the same as the subject (Price and Brady 2015). Spectral quality for ^13^C skeletal muscle data has been previously reported (Price et al. 1991, 1994a, 1994b, 1996).

### Liver MRS

Liver glycogen spectra were obtained using a modified ISIS protocol to optimize fat suppression. Subjects were positioned with an image‐guided localization MRI protocol (Gradient Echo). Each 10 min spectrum consisted of 4800 scans using a 135° pulse at coil center and a repetition time of 120 msec. ^13^C spectra were processed with a 30 Hz Lorentzian to Gaussian filter and a 500 Hz convolution difference yielding a glycogen C‐1 linewidth of 70–90 Hz (Ge et al. 2005). The resonance intensity was measured over an integrated bandwidth of 120 Hz (Petersen et al. 2004). The reproducibility of the glycogen concentration measurement has been assessed in an earlier study with a coefficient of variation between the pairs of measurements of 7% (Petersen et al. 2004). The SD in the ^13^C MRS glycogen concentration measurement due to special noise was 5% (Petersen et al. 2004).

### Statistical analysis

NMR precision was calculated by pooled variance analysis (Price et al. 1991). Because the study design created a number of subjects who could not complete the exercise protocol, subject group data were analyzed as follows: (Ahlborg et al. 1974) Each time point was first analyzed as raw data (strictly analyzed as time points so that later time points had fewer subjects). (Bemben et al. 1995) For subjects who were unable to complete the exercise protocol, data from the later time points were corrected by: (a) calculating depletion rates for subjects within the same group who completed the protocol (linear regression analysis), and (b) applying the mean calculated depletion rate to the uncompleted time points of subjects who failed to complete the protocol beginning with their last completed time point. Paired two‐tailed *t*‐tests were used for comparison of data within individual subjects. Between‐group comparisons were performed using ANOVA with Bonferroni correction factor. Data are presented as mean ± SE and significance is calculated according to *P ≤ 0.05.

## Results

In the area of performance, on average, female subjects completed significantly less of the 180 min exercise protocol than males (Males 166 ± 9 min, Females (L) 112 ± 16 min [*P* = 0.0036 vs. M], Females (F) 88 ± 16 min [*P* < 0.0001 vs. M]), representing 92%, 62% (NSD vs. M) and 49% (*P* = 0.0183 vs. M) of the protocol, respectively (Table 2). Percentages of subjects who were able to complete the protocol were: M 80%, F (L) 22% and F (F) 13%. Maximum heart rates were 192 ± 2 bpm for males and 194 ± 2 bpm for females. During the exercise protocol female subjects worked at a greater percentage of maximum heart rate (averaged over the exercise period) than males (*P* < 0.0001 F vs. M), but there was no significant difference between females exercising in different menstrual phases (M 60.3 ± 2.3% bpm_max_, F(L) 72.9 ± 4.7% bpm_max_, F (F) 74.6 ± 2.3% bpm_max_). When male and female subjects were grouped together and size was correlated with completion time, the ability to complete the protocol correlated strongly with subject height and weight, and to a lesser extent, heart rate (Table 3). When subject size was correlated with mean heart rate, subject weight was most strongly correlated (Table 3).

**Table 2:** Male and female performance during prescribed exercise protocol

Subject Performance:			
	Males	Females‐Luteal	Females‐Follicular
Exercise time completed	166 ± 9 min	112 ± 16 min**	88 ± 16 min*
Percent completed	92 ± 5%	62 ± 9%	49 ± 9%***
Mean heart rate	116 ± 4 bpm	141 ± 9 bpm	147 ± 5 bpm****
Percent of max heart rate	60.3 ± 2.3%	72.9 ± 4.7%*	74.6 ± 2.3%*

* *P* < 0.0001 F vs. M, ** *P* = 0.0036 F vs. M, **^*^ *P* = 0.0183 F vs. M, **^**^ *P* = 0.0013 F vs. M.

**Table 3:** Correlational *P*‐values according to size, heart rate, sex, and time completed

Correlation *P*‐value data:						
	Weight	Height	BMI	Mean HR	Age	Sex
Correl. w/Time Completed	0.0092	(0.0080)	NS	0.0386	NS	0.0082
Correl. w/HR	0.0061	0.0398	0.0183	—	NS	(<0.0001)

Height was the (strongest) correlation with time completed, and sex was the (strongest) correlation with heart rate.

**Figure 2:**
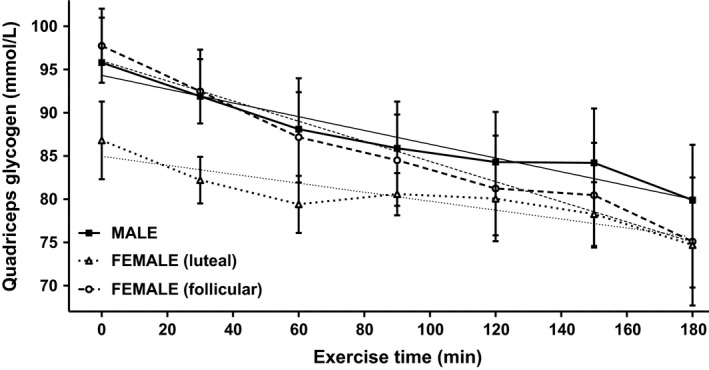
Time course of glycogen depletion in quadriceps femoris of male (■) and female (Δluteal and ○ follicular) subjects with linear regressions of depletion rates (male −4.7 ± 0.8 mmol/L‐h, female luteal −4.5 ± 2.4 mmol/L‐h, and female follicular −10.3 ± 3.5 mmol/L‐h).

**Figure 3:**
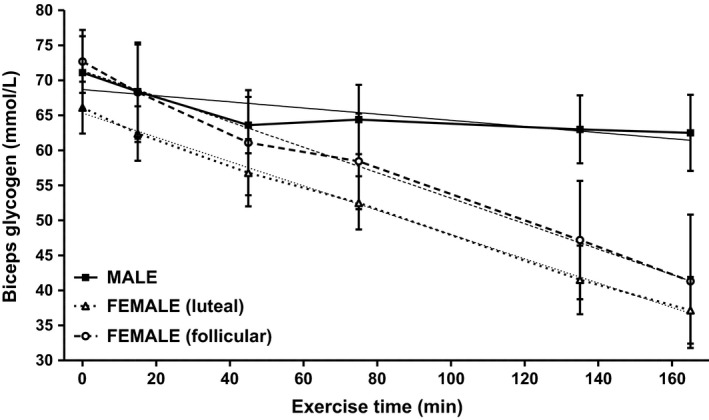
: Time course of glycogen depletion in biceps brachii of male (■) and female (Δ luteal and ○ follicular) subjects with linear regressions of depletion rates (male −2.7 ± 0.9 mmol/L‐h, female luteal −10.3 ± 1.3 mmol/L‐h, and female follicular −10.9 ± 0.5 mmol/L‐h).

While resting glycogen levels in left quadriceps femoris, measured on the day of exercise, were similar between males and females, glycogen was significantly higher in females in the follicular menstrual phase than in the luteal phase (M 95.8 ± 5.2 mmol/L, F (L) 95.0 ± 3.8 mmol/L, F (F) 81.1 ± 3.2 mmol/L [*P* = 0.0077]) (Table 4). Resting glycogen levels in the left biceps brachii were significantly lower than in the quadriceps (*P* < 0.0070) but were similar between males and females, as well as between females in different menstrual phases (M 71.1 ± 5.2 mmol/L, F (L) 65.7 ± 3.9 mmol/L, F (F) 68.5 ± 5.0 mmol/L) (Table 4). During the exercise period overall glycogen depletion rates in the quadriceps were similar between males and females and in females in different menstrual phases (M −4.7 ± 0.8 mmol/L‐h, F(L) −4.5 ± 2.4 mmol/L‐h, F(F) −10.3 ± 3.5 mmol/L‐h) (Table 4). In the biceps, overall depletion rates differed between males and females (M −2.7 ± 0.9 mmol/L‐h, F(L) 10.3 ± 1.3 mmol/L‐h [*P* = 0.0004 vs. M], F(F) −16.8 ± 4.8 mmol/L‐h [*P* = 0.0122 vs. M]). Overall biceps depletion rates were similar between females in different menstrual phases owing to the scatter which resulted from subjects who weren't able to complete the protocol. When glycogen depletion rates during the initial hour of exercise were compared with depletion rates during the subsequent 2 hours a biphasic depletion pattern was observed in the quadriceps of both males and females (Table 5, Fig. 2) In the biceps a biphasic pattern was observed in males, but females exhibited a monophasic pattern, continuing to deplete glycogen throughout the exercise period (Table 5, Fig. 3).

**Table 4:** Glycogen levels at rest and immediately following exercise, and depletion rates (A) in quadriceps femoris (B) in biceps brachialis muscles during prolonged lift/carry exercise

	Glycogen @ Rest	Total glycogen depleted	Glycogen depletion rate
(A.) Left quadriceps femoris:			
Males	95.8 ± 5.2 mmol/L	−13.1 ± 2.2 mmol/L	−4.7 ± 0.8 mmol/L‐h
Females – Luteal	81.1 ± 3.2 mmol/L*	−7.6 ± 4.0 mmol/L	−4.5 ± 2.4 mmol/L‐h
Females – Follicular	95.0 ± 3.8 mmol/L	−11.2 ± 3.1 mmol/L	−10.3 ± 3.5 mmol/L‐h
(B.)Left biceps brachialis:			
Males	71.1 ± 5.2 mmol/L#	−8.3 ± 3.7 mmol/L	−2.7 ± 0.9 mmol/L‐h
Females – Luteal	66.1 ± 3.7 mmol/L#	−16.0 ± 2.0 mmol/L	−10.3 ± 1.3 mmol/L‐h**
Females – Follicular	72.7 ± 4.5 mmol/L#	−15.9 ± 4.3 mmol/L	−16.8 ± 4.8 mmol/L‐h***
(C.)Liver:			
Males	192.9 ± 15.5 mmol/L	−59.3 ± 7.6 mmol/L	−0.178 ± 0.026 mmol/L‐min
Females – Luteal	170.3 ± 12.4 mmol/L	−28.8 ± 5.0 mmol/L****	−0.114 ± 0.022 mmol/L‐min
Females – Follicular	191.1 ± 23.7 mmol/L	−44.3 ± 16.5 mmol/L	−0.174 ± 0.063 mmol/L‐min

*P = 0.0077 FL vs. M, **P = 0.0004 FL vs. M, **^*^ *P* = 0.0122 FF vs. M. (C) Glycogen levels at rest, total amount of glycogen depleted during total time period (exercise time + rest periods), and depletion rates (**^**^ *P* = 0.0123 FL vs. M). In all test groups glycogen levels in biceps at rest were lower than in quadriceps (^#^ *P* < 0.0070).

**Table 5:** Biphasic glycogen depletion rates in quadriceps femoris and biceps brachialis muscles during prolonged lift/carry exercise

Quadriceps femoris:		
	0–60 min	60–180 min
Males	7.7 ± 2.1 mmol/L‐h	2.7 ± 1.1 mmol/L‐h*
Females – Luteal	7.9 ± 2.9 mmol/L‐h	2.3 ± 1.9 mmol/L‐h*
Females – Folicular	10.5 ± 1.7 mmol/L‐h	5.6 ± 2.1 mmol/L‐h*

Biceps brachialis:		
	0–45 min	45–165 min
Males	9.9 ± 0.3 mmol/L‐h	0.7 ± 0.4 mmol/L‐h*
Females – Luteal	12.2 ± 0.9 mmol/L‐h	10.1 ± 0.3 mmol/L‐h
Females – Folicular	15.3 ± 0.8 mmol/L‐h	10.2 ± 0.8 mmol/L‐h

*P < 0.05 early exercise vs. subsequent prolonged exercise.

Liver glycogen levels at rest were similar between males and females, and between females in different menstrual phases (M 192.9 ± 15.5 mmol/L, F (L) 170.3 ± 12.4 mmol/L, F (F) 191.1 ± 23.7 mmol/L). Liver glycogen depletion rates during exercise were not significantly different from depletion rates at rest in both sexes as well as within females in different menstrual phases (M −0.178 ± 0.025 mmol/L‐min, F(L) −0.114 ± 0.022 mmol/L‐min, F(F) −0.174 ± 0.063 mmol/L‐min) (Table 4). In females during the luteal phase net liver glycogen concentrations exhibited significantly less decline over the entire experimental period (preparation period + exercise periods + rest periods) than males (*P* = 0.0132 FL vs. M); however, no differences existed between subjects in luteal and follicular phases (Table 4).

## Discussion

The essential finding of this study was that women were not able to complete the exercise protocol as well as men, and that this performance difference is strongly correlated with the size (weight, height, BMI) of the individual subject. The correlation also carried over to size versus workload (measured as mean heart rate). When muscle and liver glycogen metabolism was studied as a function of sex (M vs. F) and menstrual cycle variations (within the female group), there were some significant differences and a couple of trends that approached significance. While resting glycogen levels in the quadriceps were similar between males and females in the follicular menstrual phase, females stored significantly less glycogen during the luteal phase than males. This difference was not seen in the biceps; however, resting biceps glycogen levels were significantly lower than in the quadriceps in all groups.

During exercise quadriceps glycogen depletion rates in females (follicular phase) trended toward significance when compared with depletion rates in males and females (luteal phase). In addition, there was a trend toward less total exercise‐induced glycogen depletion in females in the luteal phase. While these differences are not significant, owing primarily to the small sample size, they are indicative of an important trend toward glycogen sparing during the luteal phase of the menstrual cycle. In the upper body females depleted biceps glycogen at a significantly higher rate than males but did not differ according to menstrual phase, perhaps due to the greater workload challenge to women. Liver glycogen depletion rates were not significantly greater during exercise as compared to normal depletion rates at rest (Petersen et al. 2004); however, females in the luteal group depleted significantly less total glycogen than males. Taken together, the results of this study suggest that there are systemic mechanisms which work in concert to accommodate size differences and spare carbohydrate reserves during prolonged lift/carry exercise in men and women and that menstrual variations play a role.

The correlations between size and performance suggest that in this study size, rather than sex, was the major controlling variable. Because of their overall smaller size, the females in both menstrual phases worked harder (according to mean HR) and failed to complete the protocol more often than their male counterparts. As in this study, most of the previous studies comparing upper/lower body strength have studied elbow flexors (biceps brachii) and knee extensors (quadriceps femoris) (Maughan et al. 1983; Miller et al. 1993; Kanehisa et al. 1994; Sharp 1994).

Studies comparing size and strength have reported female biceps CSA's at 55% of males and quadriceps CSA's at 75% of males, whereas upper body strength was 50–60% of males and lower body strength was 66–68% of males (Miller et al. 1993; Sharp 1994) although not all data are in agreement (Maughan et al. 1983; Kanehisa et al. 1994). These male/female performance results (percent completion of the protocol) are in agreement with the previous studies with female participants performing at about 60% of male participants. However, predicting male/female performance is likely influenced by other factors such as menstrual cycle (Jurkowski et al. 1981; Hicks et al. 2001). In this study, female follicular performance time was significantly lower than males, but luteal performance was not. Women also worked at a significantly higher percent of HR_max_ than men, suggesting that the challenge to the upper body was a significant factor in performance.

Before beginning to exercise male and female resting levels of muscle glycogen in the quadriceps were not significantly different; however, female follicular resting glycogen was greater than that of female luteal. This observation is in agreement with previous studies that have noted greater glycogen utilization with exercise during the follicular phase as compared with the luteal phase (Lavoie et al. 1987; Zderic et al. 2001; Devries et al. 2006), suggesting that the body stores more glycogen in heavily used muscles during the follicular phase in order to be prepared for a potential challenge. In the biceps, resting glycogen levels were not significantly different between males and females, nor were they different in the different menstrual phases. In all groups studied resting glycogen levels in the quadriceps were significantly greater than levels in the biceps (*P* = 0.0061 M, *P* = 0.0062 FL, *P* = 0.0012 FF). This observation would seem to support the notion that, at rest, the body selectively stores carbohydrate reserves, perhaps based upon muscle utilization patterns.

During exercise, overall quadriceps glycogen depletion rates were not significantly different between males and females, nor were they different between menstrual phases. While it is difficult to compare data from studies of an isolated muscle during exercise with this study which employs a systemic protocol, these results suggest that in both men and women the quadriceps muscles were not heavily challenged (Price et al. 1991, 1994a). When quadriceps glycogen depletion rates, assessed over the first hour of exercise, were compared with depletion rates during the subsequent two hours of exercise, a biphasic depletion pattern was observed. While the depletion pattern resembled those seen in our earlier studies, depletion slowed significantly but did not cease as previously observed (Price et al. 1991, 1994a). Again, this may be the result of attempting to compare an isolated muscle exercise protocol with a systemic protocol, in which we cannot account for the contributions of other muscles. It is also possible that over the 3 h exercise period employed in this study total glycogen was not depleted to the cessation point of about 20 mmol/kg‐ww observed in the 1991 study (Price et al. 1991). This suggests that, while the comparison is admittedly tenuous, in this study quadriceps were likely working at less than 20% capacity in all groups studied (Price et al. 1991).

Males and females (L) produced similar glycogen depletion rates; however, females (F) produced glycogen depletion rates that were faster, although not significantly so. This may be the result of the large scatter that resulted from the number of subjects who were unable to complete the protocol and suggests the need for further study of the menstrual cycle effect upon prolonged resistance exercise (Jurkowski et al. 1981; Lavoie et al. 1987; McCracken et al. 1994; Zderic et al. 2001; Devries et al. 2006).

Overall biceps glycogen depletion rates were significantly greater in women during exercise as compared with men; however, there was no significant difference between women in different menstrual phases. The observed glycogen depletion rates suggest that, while the biceps were not heavily challenged in men, the women were working their biceps at a higher rate. Comparing glycogen depletion rates during the first 45 min of exercise versus the subsequent 2 h produced a biphasic depletion pattern in the males, but not in the females. The male depletion pattern suggests that the men were working at about 25% of MVC and that glycogen depletion ceased after 45 min; however, total glycogen depletion did not reach the levels seen in the 1991 study suggesting again the difficulty in comparing an isolated muscle study with a systemic study (Price et al. 1991). In the females, during both menstrual phases, the glycogen depletion pattern was roughly monophasic not slowing appreciably from its initial rate during the final 2 h of exercise. This result is more in agreement with our studies of glycogen recovery following a predepletion exercise protocol in which subjects manipulated their body weight until 75% of gastrocnemius glycogen was depleted (Price et al. 1994b, 1996). Workloads in those earlier studies were 49–55% of MVC and glycogen depletion rates were >100 mmol/L‐h (Price et al. 1994b, 1996). Female glycogen depletion rates in this study were not as great, but over the course of the exercise protocol glycogen depletion rates failed to decrease significantly, suggesting that the women were working their biceps at >30% of MVC. It should be noted again that these estimated %MVC values are tentative owing to our attempt to compare different exercise protocols; however, we feel that this explanation of our current data represents a rough approximation of the overall physiological events that are supporting the complex load‐bearing exercise being performed by these subjects.

Net liver glycogen depletion rates, measured at rest prior to the beginning of exercise and over the course of the exercise session, were not significantly increased by the exercise. This is in disagreement with our earlier study (Petersen et al. 2004); however, resting rates were in agreement with previously reported rates (Petersen et al. 2004). Net depletion rates were not significantly different between men and women, nor were they significantly different between women in different menstrual phases. These results, while different from the earlier study of treadmill running which observed a significant increase in glycogen depletion rates at both 35% (0.5213 mmol/L‐min) and 70% of VO_2max_ (0.7975 mmol/L‐min) (Petersen et al. 2004), are likely a result of two factors: (1) different exercise protocols resulting in, (2) different metabolic responses. The number of muscles responding to the current load‐bearing lift/carry exercise and how hard they are working is unknown, and may account for a significant production of metabolic intermediates such as lactate and glycerol. If these are present in the bloodstream in significant amounts and being taken up by the liver, gluconeogenesis may be contributing glycogen (Wahren et al. 1971; Ahlborg et al. 1974; Rooney et al. 1993). This would result in a lower net glycogen depletion rate, in agreement with animal data (Wasserman et al. 1989; Rooney et al. 1993). Human and animal studies have shown that lipid mobilization is increased in the presence of estrogen as is seen during the luteal phase (Benoit et al. 1982; Ellis et al. 1994; Casazza et al. 2004).

This study observed exercising liver glycogen rates during the luteal phase that were 34% lower than rates during the follicular phase (and 36% lower than males), although they were not established as significant. However, total net glycogen depletion was significantly less in females (luteal phase) than in males suggesting that gluconeogenesis is increased in women during the luteal phase of their menstrual cycle. This points to the need for further investigation of this metabolic phenomenon.

The intention of this study was to compare responses to an identical non‐normalized workload in three different groups: (1) men, (2) women in their luteal menstrual phase, and (3) women in their follicular menstrual phase. These conditions are similar to those that might be encountered by male and female employees in the workplace. When men and women are called upon to perform prolonged lifting and carrying exercise, as is required of many day‐to‐day tasks in a variety of occupations, the systemic energy response is distributed throughout the body. In this study, we have used ^13^C NMR spectroscopy to demonstrate that the male and female responses to a prolonged non‐normalized lift/carry exercise protocol are different, and that differences also exist between women who are exercising during the luteal phase of their menstrual cycle versus during the follicular phase.

We conclude the following: (1) Based upon body size, women are unable to complete as much of a three hour non‐normalized lift/carry exercise protocol as men. (2) During exercise women must work harder to perform less total work. (3) Glycogen storage in the quadriceps at rest is similar between women in the follicular menstrual phase and men, but significantly less in women during the luteal menstrual phase. (4) Male/female glycogen storage is similar in the biceps and the liver. (5) Men and women utilize stored quadriceps carbohydrates at similar rates, but women utilize stored biceps carbohydrates at a faster rate than men.

## Conflict of Interest

None declared.
